# Evaluation of two commercial diagnostic methods for HHV-8 viral load assessment

**DOI:** 10.1016/j.ijregi.2024.100374

**Published:** 2024-05-06

**Authors:** Honorine Fenaux, Lina Mouna, Corinne Vieux-Combe, Isabelle Thouard, Philippe Colliot, Anne-Marie Roque-Afonso

**Affiliations:** 1 AP-HP, Hôpital Paul Brousse, Laboratoire de Virologie, Villejuif, France; 2 Université Paris-Saclay, Inserm U1993, AP-HP Hôpital Paul Brousse, Villejuif, France

**Keywords:** HHV-8, Viral load, Quantification, Follow-up

## Abstract

**Objectives:**

Human herpesvirus-8 (HHV-8) can cause Kaposi's sarcoma or B lymphoproliferative disorders such as multicentric Castleman disease. Patient follow-up is based on assessing the HHV-8 viral load, which is usually achieved using real-time polymerase chain reaction (PCR). The HHV-8 Premix r-gene kit (BioMérieux) was used by some laboratories in the past, but BioMérieux ceased the production and distribution of this kit in 2021-2022. Other kits need to be tested so that they can be used for diagnostic purposes. Here we evaluated two commercial kits: HHV8 ELITe MGB Kit (ELITech) and Quanty HHV-8 (Clonit) and compared them with the HHV-8 Premix r-gene kit.

**Methods:**

We used whole blood samples that had previously been tested with the HHV-8 Premix r-gene kit for diagnostic purposes. Overall, 46 samples (37 HHV-8-positive and 9 HHV-8-negative) were tested with the ELITe MGB Kit and 37 (29 HHV-8-positive and 8 HHV-8-negative) with the Quanty HHV-8 kit. The different methods were compared using Bland-Altman and Passing-Bablok tests with Analyse-it software.

**Results:**

Qualitative results were concordant except for one HHV-8 low-positive sample that was found to be negative by the ELITe MGB Kit. The quantitative results were also concordant; both kits showed mean differences of 0.58 log_10_ copies/ml and 0.73 log_10_ copies/ml, respectively, compared to the Premix r-gene kit.

**Conclusions:**

Both the methods tested produced acceptable results and could be used for diagnostic purposes. It should be remembered that there is no international standard for HHV-8 quantification and that patients should always be followed using the same method.

## Highlights

• Performances are acceptable for both methods in a diagnostic context.
• Both methods are automatable on a QiaSymphony SP/AS platform and Rotor-Gene Q.
• There is no international standard for human herpesvirus-8 viral load quantification.
• A patient should always be followed with the same method.

## Introduction

Human herpesvirus-8 (HHV-8) belongs to the Herpesviridae family and the Gammaherpesvirinae subfamily. It is mostly transmitted via saliva [1], although vertical, sexual, and blood and organ transplantations are other possible routes [1]. Its seroprevalence differs around the world: it is around 2-4% in Europe, Southeast Asia, and the Caribbean, about 10% in Mediterranean populations and around 40% in Sub-Saharan Africa [2]. It can cause Kaposi's sarcoma or B lymphoproliferative disorders such as multicentric Castleman disease [3].

Patient follow-up is based on assessing the HHV-8 viral load as this has been shown to correlate with disease progression or regression [[4], [5], [6]]. The method usually employed to assess HHV-8 viral load is real-time polymerase chain reaction (PCR), which in France is only performed by a few laboratories. There are no quantification standards and different methods can produce markedly varying quantitative results. In the past, the HHV-8 Premix r-gene kit (BioMérieux, Marcy-l’Étoile, France) was used in some laboratories (including ours) but BioMérieux ceased production and distribution of this kit in 2021-2022. Other kits therefore need to be tested so that they can be used for diagnostic purposes.

## Objectives

Here we evaluated two commercial kits: HHV8 ELITe MGB Kit (ELITech, Puteaux, France) and Quanty HHV-8 (Clonit, Abbiategrasso, Italy) and compared them with the HHV-8 Premix r-gene kit (BioMérieux, Marcy-l’Étoile, France), which is no longer in production. These tests are referred to as ELITech, Clonit, and Argene, respectively, in the text that follows.

## Study design

### Samples

Whole blood samples collected on Ethylenediamine tetraacetic acid (EDTA) tubes, and external quality assessment controls that had previously been frozen at -80°C, were assessed. They had been tested using the Argene kit for diagnostic purposes. We tested them with the ELITech or Clonit assays but did not retest them with our reference method for feasibility reasons.

### Analytical procedure

The HHV-8 PCR tests were performed on whole blood and were adapted to the QiaSymphony SP/AS platform and Rotor-Gene Q (Qiagen, Hilden, Germany). Nucleic acids were extracted using the QIAsymphony DSP deoxyribonucleic acid (DNA) mini kit (Qiagen).

### Reference assay: HHV-8 Premix R-gene™ (BioMérieux)

The Argene kit enabled the amplification of a 146 bp-fragment on the open reading frame 26 (ORF26) gene (encoding the minor capsid protein). The internal control DNA Internal Control (DICO) (BioMérieux) was added before extraction and amplified in a separate well. The kit was used according to the manufacturer's instructions. Production of this kit was halted in 2021.

### HHV8 ELITe MGB Kit (ELITech)

The ELITech kit enables amplification of a fragment of the ORF26 gene. A fragment of the gene encoding human beta-globin is amplified in the same well as an internal control. According to the manufacturer, analytical sensitivity with a 95% probability of detection reaches 117 gEq/mL.

Positive (37) and negative (9) whole blood samples previously tested using the HHV-8 Argene kit were evaluated retrospectively. These nine HHV-8 negative whole blood samples produced positive results for other Herpesviridae (2 herpes simplex virus (HSV)-1, 1 varicella zoster virus (VZV), 1 cytomegalovirus (CMV), 1 CMV + Epstein-Barr virus (EBV), 2 HHV-6A, 2 EBV + HHV-6B).

We also tested three quality control plasma samples (Quality Control for Molecular Diagnostics, Glasgow, United Kingdom) which were positive for HSV-2 (n = 2) or VZV (n = 1) and presumed negative for HHV-8.

A highly positive sample (232,000 cp/ml in ELITech) was serially diluted 10-fold up to 1:100,000 and PCR HHV-8 was performed at each dilution (in triplicate for the last three: 1:1,000, 1:10,000 and 1:100,000) to assess the analytical sensitivity of the method.

### Quanty HHV-8 (Clonit)

The Clonit kit enables amplification of a fragment of the ORF26 gene. A fragment of the gene encoding human beta-globin is amplified in the same well as an internal control. According to the manufacturer, analytical sensitivity with a 95% probability of detection reaches 567 cp/ml.

Because an insufficient sample volume was available, it was not possible to test some samples with both ELITech and Clonit.

We tested 29 whole blood samples that had been found to be PCR HHV-8 positive with Argene (27 of which had also been tested with ELITech), and eight whole blood samples that were negative for PCR HHV-8 with Argene but positive for other Herpesviridae (2 HSV-1, 1 VZV, 1 CMV + EBV, 1 HHV-6A, 2 EBV + HHV-6B, 1 CMV + HHV-6B).

A highly positive sample (1,318,830 cp/ml in Clonit) was serially diluted 10-fold up to 1:100,000 and PCR HHV-8 was performed at each dilution (six times at dilution 1:1,000, which was very close to the limit of detection, and in triplicate for the last two, 1:10,000 and 1:100,000) to assess the analytical sensitivity of the method.

### Statistics

Comparisons between the different methods were performed using the Bland-Altman and Passing-Bablok tests, with Analyse-it software.

## Results

### *Comparison between* ELITech *and Argene*

Among the 37 positive samples, 36 were positive with ELITech (clinical sensitivity: 97%). The sample for which the results were conflicting was the lowest positive (740 copies/ml in Argene). All negative samples were found to be negative with ELITech (specificity: 100%).

Analytical sensitivity was calculated by means of serial 10-fold dilutions of a highly positive sample and found to be 150 copies/ml.

**Figure 1:**
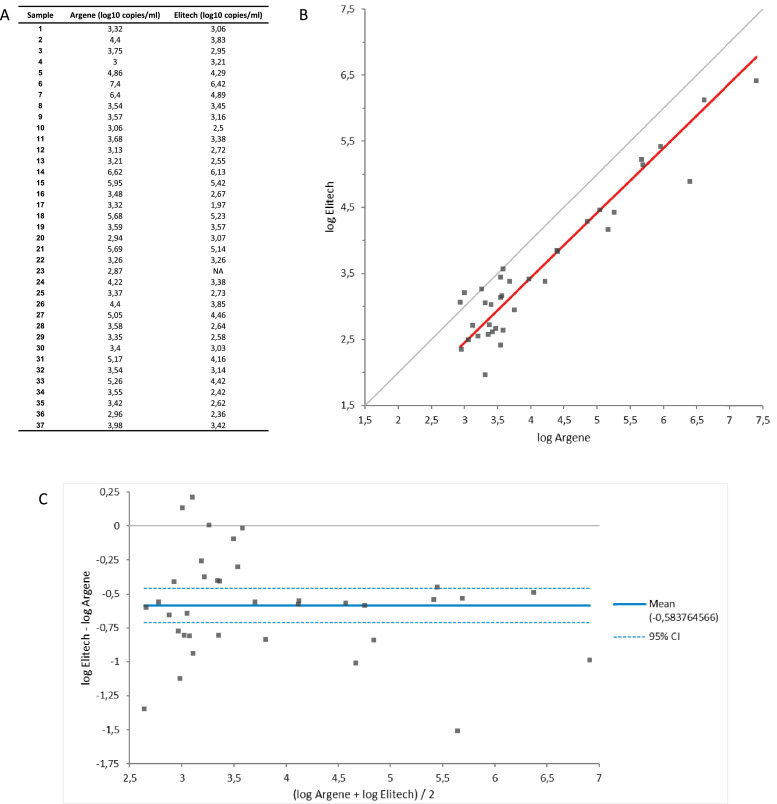
Results of comparison between HHV8 ELITe MGB Kit (ELITech) and HHV-8 Premix r-gene (BioMérieux). (A) result in log_10_ copies/ml for each sample tested. (B) Correlation curve, Passing-Bablok (y = -0.4857 + 0.9805 x). (C) Bland-Altman plot.

The comparison of quantitation methods was concordant (r = 0.948) and revealed a mean difference of 0.58 log_10_ copies/ml, with a standard deviation of 0.38 (Figure 1).

**Figure 2:**
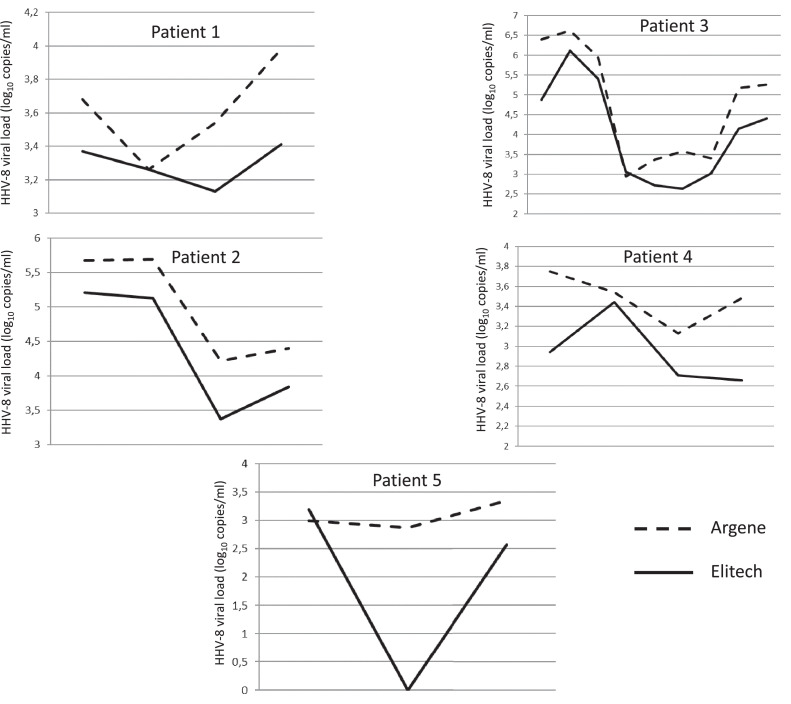
Longitudinal follow-up of five patients using the Argene and ELITech methods. The second sample from patient 5 (low positive with Argene) produced a contrasting result with ELITech. Underlying HHV-8 condition: patient 1–Kaposi's sarcoma, patient 2–multicentric Castleman disease, patient 3–multicentric Castleman disease, patient 4–Kaposi's sarcoma, patient 5–information not available.

The test was used to follow the viral loads of five patients over time (Figure 2) and produced concordant results.

### Comparison between Clonit and Argene

All positive samples were found to be positive with Clonit (clinical sensitivity: 100%). All negative samples were found to be negative with Clonit (specificity: 100%).

Analytical sensitivity was calculated by means of serial 10-fold dilutions of a highly positive sample and found to be 2862 copies/ml.

**Figure 3:**
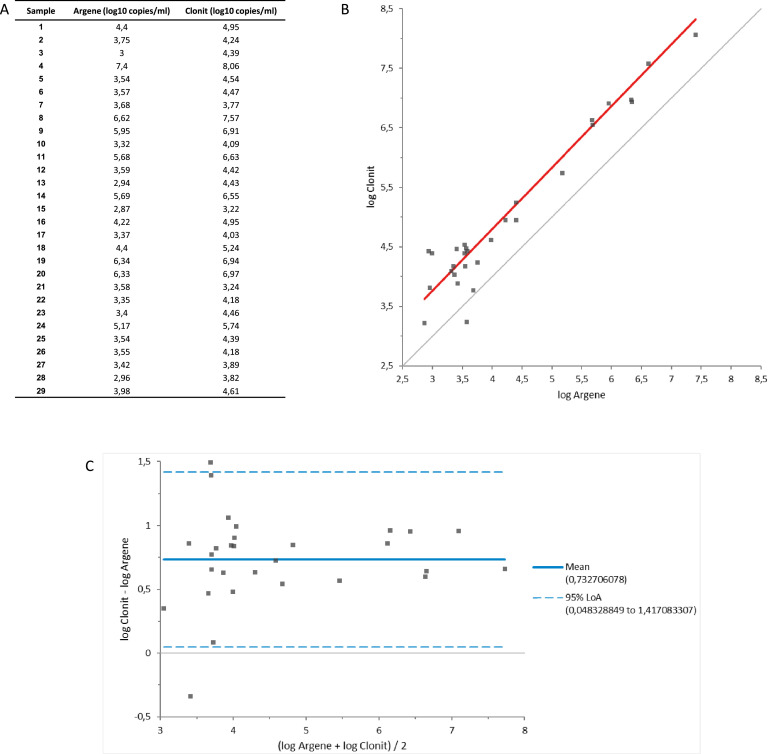
Results of comparison between Quanty HHV-8 Kit (Clonit) and HHV-8 Premix r-gene (BioMérieux). (A) result in log_10_ copies/ml for each sample tested. (B) Correlation curve, Passing-Bablok test (y = -0.6526 + 1.037 x). (C) Bland-Altman plot.

The comparison of quantitation methods was concordant (r = 0.965) and revealed a mean difference of 0.73 log_10_ copies/ml, with a standard deviation of 0.35 (Figure 3).

**Figure 4:**
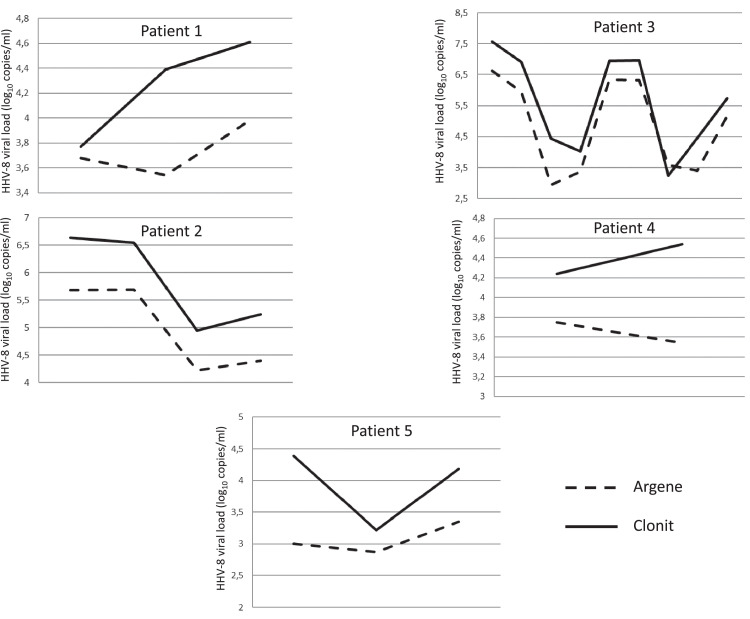
Longitudinal follow-up of five patients using the Argene and Clonit methods. Underlying HHV-8 condition: patient 1–Kaposi's sarcoma, patient 2–multicentric Castleman disease, patient 3–multicentric Castleman disease, patient 4–Kaposi's sarcoma, patient 5–information not available.

The test was used to follow the viral loads of five patients over time (Figure 4) and produced concordant results.

## Discussion

During this study we compared two commercial kits for HHV-8 quantitative PCR assays that both produced acceptable performances and can be used for diagnosis. Both can also be automated on a QiaSymphony SP/AS platform and Rotor-Gene Q.

Quantitative analysis demonstrated agreement between the findings obtained with the ELITe MGB and Quanty HHV-8 kits, which displayed mean differences of 0.58 log_10_ cp/ml and 0.73 log_10_ cp/ml, respectively when compared to the Premix r-gene kit.

Indeed, there is currently no standard for HHV-8 quantification, and patients should always be followed using the same method so that any changes to viral loads can be interpreted. Consequently, the analytical sensitivity thresholds could not be compared between ELITech and Clonit as the number of copies per milliliter did not match between these different methods.

The Clonit and Argene tests had already been compared versus an in-house method and this showed that the findings of Argene were closer to those of the reference method employed [7]. Clonit overestimated the viral load compared with the in-house PCR and produced positive results for samples that were undetectable using the in-house PCR. We were not able to obtain such results with Clonit. The result found by the previous authors might possibly have been because of a difference in sensitivity between Clonit and their in-house method.

We compared the follow-up of five patients using each method and found that the kinetics were about the same and would not have led to any changes to the medical care received by these patients.

One limitation of our study is that the samples were not reassessed using the reference method at the same time as they were used to test another method; we used the values found at the first assay, that is, at the time of diagnosis. The samples had thus undergone at least one freezing/thawing cycle between the tests using the two methods. However, testing both tests on the same day was not feasible because the QiaSymphony SP/AS platforms were already used every day for diagnostic purposes that could not be delayed (notably to detect cytomegalovirus and Epstein-Barr virus).

In conclusion, the commercial kits during this study exhibited acceptable performance in terms of HHV-8 detection for diagnostic purposes. However, it is essential to note the current absence of an international standard for HHV-8 quantification, underscoring the importance of consistent methodological approaches for patient follow-up. Discontinuation of the HHV-8 Premix r-gene kit has emphasized the ongoing need to evaluate and validate alternative assays to ensure continued quality for HHV-8 detection and monitoring.

## Declarations of competing interest

The authors have no competing interests to declare.
